# Inflammatory Myofibroblastic Tumor of the Bladder: 2 Rare Cases Managed with Laparoscopic Partial Cystectomy

**DOI:** 10.1155/2016/4976150

**Published:** 2016-11-15

**Authors:** Sofia Santos Lopes, Andrea Furtado, Rita Oliveira, Ana Cebola, Bruno Graça, Manuel Ferreira Coelho, Fernando Ferrito, Carrasquinho Gomes

**Affiliations:** ^1^Urology Department, Hospital Prof. Dr. Fernando Fonseca, IC 19, Venteira, Amadora, Portugal; ^2^Pathology Department, Hospital Prof. Dr. Fernando Fonseca, IC 19, Venteira, Amadora, Portugal

## Abstract

Two cases of inflammatory myofibroblastic tumor (IMT) of the bladder are reported here. Both patients were male and presented with macroscopic hematuria; in the first case terminal hematuria was associated with irritative voiding symptoms. The second case was a smoker with hematuria unresponsive to medical treatment and anemia. Clinical presentation, pathological features, treatment, and prognosis are discussed. Due to rarity of this pathological condition, there are no guidelines concerning treatment and follow-up. We present our follow-up scheme and highlight the use of laparoscopic partial cystectomy as a successful treatment approach.

## 1. Introduction

Inflammatory myofibroblastic tumor of the bladder was first described in 1980 by Roth [1]; it is a rare benign myofibroblastic proliferation that has been given several designations, including inflammatory pseudotumor, inflammatory pseudosarcomatous fibromyxoid tumor, nodular fasciitis, pseudosarcomatous myofibroblastic tumor, and fibromyxoid pseudotumor [2].

Most of the patients are young, with a mean age of 38.9 years and a slightly female predominance of 51.7% in some studies [3], while others report a male predominance of 9 : 8 [4]. The most common symptom is hematuria, followed by irritative or obstructive voiding symptoms and lower abdominal pain [2, 3, 5]. Tumor size descriptions range from 1.5 to 13 cm [2]. IMT etiology is still unknown; prior history of surgery or instrumentation, trauma, and steroid use has been associated with it but due to its rarity it remains uncertain [3, 5].

Herein, we report two cases with different presentations of this rare clinical entity.

## 2. Case Presentation

**Figure 1:**
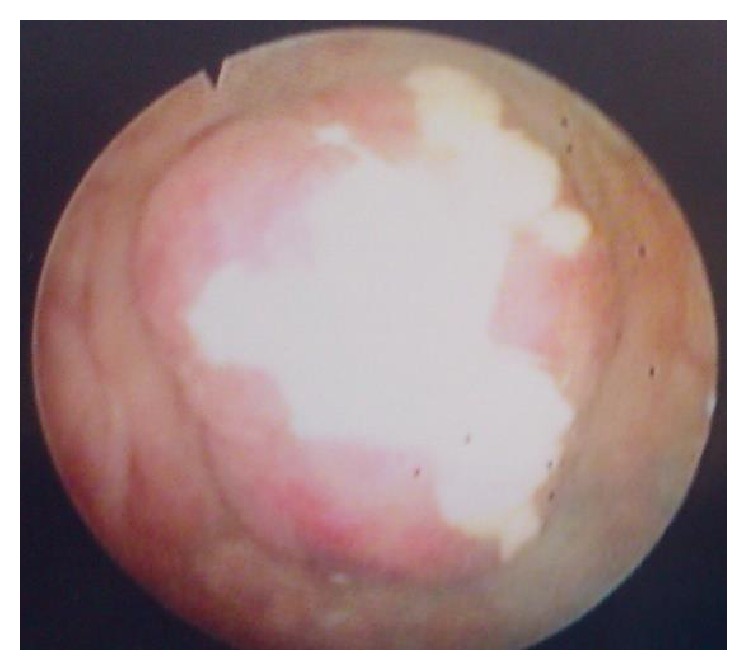
First case tumor cystoscopic appearance.

**Figure 2:**
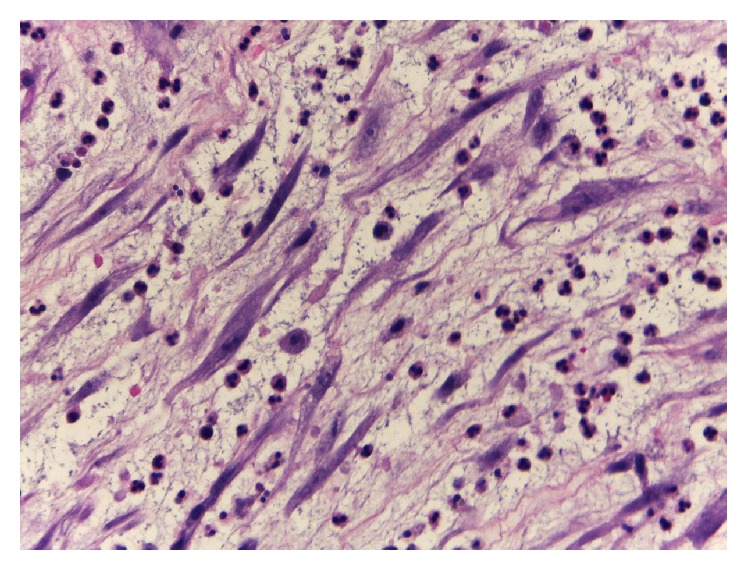
Histological examination (hematoxylin-eosin staining; magnification 10 × 40), showing spindle cell proliferation with a fascicular pattern admixed with inflammatory cells on a myxoid background.

**Figure 3:**
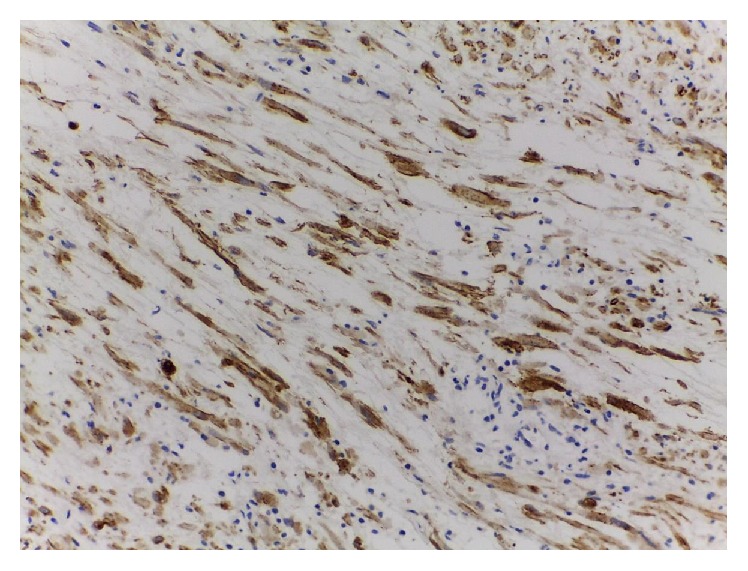
Histological examination of the second case (magnification 10 × 20), positive ALK-1 staining.

**Figure 4:**
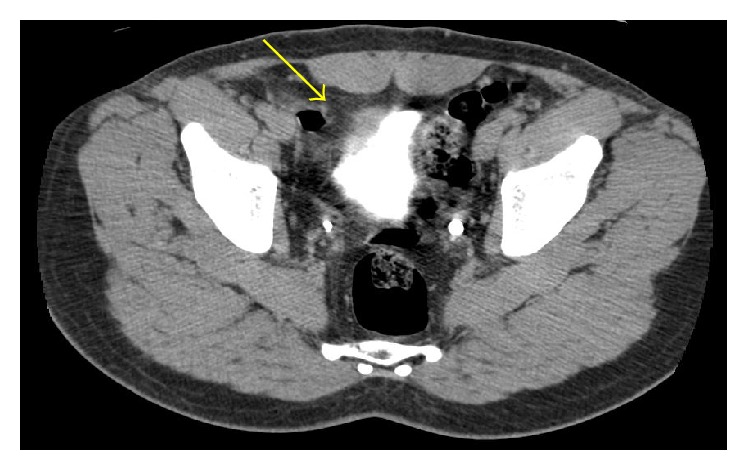
CT-scan of the second case showing a bladder wall thickening, with contrast enhancement.

(1) A 38-year-old nonsmoking male presented with burning micturition and terminal macroscopic hematuria for one month period, with no other symptoms or previous relevant medical history. Physical exam and routine blood profile were normal. Pelvic ultrasound revealed an asymmetrical thickening of the right anterolateral bladder wall (maximum thickness 23.5 mm). Cystoscopy showed a well-defined solid lobulated lesion with a fibrinous exudate (Figure 1). It was biopsied and sent to the Pathology Department. Microscopic examination revealed spindle cell proliferation with a fascicular pattern admixed with inflammatory cells on a myxoid background (Figure 2). The immunohistochemistry study was positive for vimentin, smooth muscle actin, and ALK-1 and negative for desmin, PS100, and CD34. The diagnosis was an IMT of the urinary bladder. On computed tomography (CT) scan no local or lymph nodes invasion was detected. Laparoscopic partial cystectomy was performed, with a total duration of 100 minutes, minimal blood loss, and no perioperative complications. The histopathological examination confirmed the initial diagnosis, the tumor had 3.2 cm of largest diameter, and margins were negative. Pelvic CT at 56 months after surgery was also negative. At 58 months (4.8 years) after surgery the patient is asymptomatic, with no frequency or dysuria, and has no evidence of recurrence. Follow-up has been made with biannual cystoscopy.

(2) A 56-year-old male, with a 50-pack year history of smoking, was admitted from the urgency department for painless gross hematuria with clots. In blood tests, anemia (Hemoglobin 8.6 g/dL) was detected, no other abnormalities. Urgent transurethral bladder resection/fulguration (TUBR) was proposed to control the hematuria and remove bladder clots, where cystoscopy showed a solid and wide base/sessile lesion of the anterior wall. According to the histomorphological features and the immunohistochemical profile (Figure 3), similar to the first case, the histopathological diagnosis was an IMT of the urinary bladder with* muscularis mucosa* infiltration. CT showed a 6 cm right anterolateral bladder wall thickness, with involvement of the perivesical fat; no lymph nodes or other lesions were identified (Figure 4). Laparoscopic partial cystectomy was proposed to achieve total resection. The partial cystectomy, combined with intraoperative cystoscopy to identify mucosal abnormalities, was done in 160 minutes without perioperative complications. The tumor margins were negative. At 14 months of follow-up, CT and cystoscopy are free of recurrence signs. The patient has no storage or voiding symptoms and accomplished a smoking cessation program with success.

## 3. Discussion

Clinically and radiologically, IMT of the urinary bladder is indistinguishable from other entities, it has a broad differential diagnosis ranging from reactive to neoplastic malignant lesions, comprising postoperative spindle cell nodule, embryonal rhabdomyosarcoma, leiomyosarcoma or sarcomatoid urothelial carcinoma [2, 6]. When compared to urothelial bladder carcinoma, the most frequent bladder tumor, IMT usually presents at younger age and spares the trigone [7]. Ultrasound findings are unspecific, and on CT IMT can present as intraluminal polypoid or submucosal mass, with variable density and, usually, early peripheral enhancement [7, 8]. Perivesical fat stranding can also be present [8].

The final and definitive diagnosis can only be made by histopathological examination and immunohistochemical/molecular study, with IMT expressing mainly smooth muscle actin, desmin, and anaplastic lymphoma kinase (ALK-1) [9]. Positive cytoplasmic immunostaining for ALK has been identified in up to 89% of cases of IMT in the bladder [2]. In a recent systematic review the ALK was positive in 65% of 120 patients analyzed [3], but no conclusions where possible about the difference between ALK positive or negative IMT of the bladder.

Despite being a benign lesion it can recidivate locally, possibly due to muscularis mucosa invasion, described in 41% of the patients in a review by Montgomery et al. [9]. At least one case of malignant IMT of the bladder with local recurrence and multiple lymph node, bone, and soft tissue metastases was reported [5].

There are few case reports concerning conservative therapy in bladder IMT; in one case a successful reduction in the size of a bladder IMT with anti-inflammatory regime (prednisone and Cox-2 inhibitor) was achieved [10], while others reported complete conservative treatment using ibuprofen and amoxicillin/clavulanate potassium [11].

Complete local resection with negative surgical margins at pathology is the more advisable treatment and the most important prognostic factor, which can be achieved by transurethral resection (only if superficial) or partial cystectomy. Concerning the possibility of recurrence it is our opinion that partial cystectomy is more effective in total removal of the lesion, as presented in this paper.

The first description of the laparoscopic approach in partial cystectomy for IMT was done by Pradhan et al. in 2013 [12]; in our two cases we used a similar technique since both tumors were located anteriorly without need for ureteral mobilization. In Trendelenburg position, 4 transperitoneal ports were introduced and, after bladder dissection, the tumor was identified using the flexible cystoscope light and a security margin was left. The bladder was closed in two layers using Vicryl® 0, a Foley catheter 16 Fr was left for ten days, and a pelvic drain was left for 48 hrs. Our patients were discharge after pelvic drain removal, at second postoperative day, with no complications.

Laparoscopic partial cystectomy can play an important role in the management of this rare bladder tumor, allowing complete resection with few comorbidities and rapid recovery in a pathology that mainly affects young patients.

Regarding follow-up, there are no standardized schemes but it is advisable since this tumor has 25% of recurrence [7]. We adopt biannual urethrocystoscopy and CT every 2 years for the first 5 years. One of the patients is now reaching five years of follow-up and we are planning to change to annual urine sample analysis and pelvic ultrasonography evaluation.

## References

[1] Roth J. A. (1980). Reactive pseudosarcomatous response in urinary bladder. *Urology*.

[2] Lott S., Lopez-Beltran A., MacLennan G. T., Montironi R., Cheng L. (2007). Soft tissue tumors of the urinary bladder, part I: myofibroblastic proliferations, benign neoplasms, and tumors of uncertain malignant potential. *Human Pathology*.

[3] Teoh J. Y. C., Chan N.-H., Cheung H.-Y., Hou S. S. M., Ng C.-F. (2014). Inflammatory myofibroblastic tumors of the urinary bladder: a systematic review. *Urology*.

[4] Wei L., Jianbo L., Quiang W., Hai Y., Zhixiang L. (2013). Inflammatory myofibroblastic tumor of the bladder: case report and review of the literature. *Canadian Urology Association Journal*.

[5] Kim H. W., Choi Y. H., Kang S. M. (2012). Malignant inflammatory myofibroblastic tumor of the bladder with rapid progression. *Korean Journal of Urology*.

[6] Süer E., Gülpnar Ö., Mermerkaya M. (2012). Inflammatory myofibroblastic tumor of the bladder in a 10-year-old girl. *Urology*.

[7] Rosado E., Pereira J., Corbusier F., Demeter P., Bali M. A. (2015). Inflammatory pseudotumor of the urinary bladder. *The Journal of Radiology Case Reports*.

[8] Surabhi V. R., Chua S., Patel R. P., Takahashi N., Lalwani N., Prasad S. R. (2016). Inflammatory myofibroblastic tumors. *Radiologic Clinics of North America*.

[9] Montgomery E. A., Shuster D. D., Burkart A. L. (2006). Inflammatory myofibroblastic tumors of the urinary tract: a clinicopathologic study of 46 cases, including a malignant example inflammatory fibrosarcoma and a subset associated with high-grade urothelial carcinoma. *American Journal of Surgical Pathology*.

[10] Berger A., Kim C., Hagstrom N., Ferrer F. (2007). Successful preoperative treatment of pediatric bladder inflammatory myofibroblastic tumor with anti-inflammatory therapy. *Urology*.

[11] Fletcher S. G., Galgano M. T., Michalsky M. P., Roth J. A. (2007). Regression of inflammatory pseudotumor of the bladder in a child with medical management. *Urology*.

[12] Pradhan M. R., Ranjan P., Rao R. N., Chipde S. S., Pradhan K., Kapoor R. (2013). Inflammatory myofibroblastic tumor of the urinary bladder managed by laparoscopic partial cystectomy. *Korean Journal of Urology*.

